# Selection of *DDX5 *as a novel internal control for Q-RT-PCR from microarray data using a block bootstrap re-sampling scheme

**DOI:** 10.1186/1471-2164-8-140

**Published:** 2007-06-01

**Authors:** Li-Jen Su, Ching-Wei Chang, Yu-Chung Wu, Kuang-Chi Chen, Chien-Ju Lin, Shu-Ching Liang, Chi-Hung Lin, Jacqueline Whang-Peng, Shih-Lan Hsu, Chen-Hsin Chen, Chi-Ying F Huang

**Affiliations:** 1 Institute of Cancer Research, National Health Research Institutes, Taipei 114, Taiwan; 2 Department of Surgery, Veterans General Hospital, Taipei 112, Taiwan; 3 Institute of Statistical Science, Academia Sinica, Taipei 115, Taiwan; 4 Department of Chemical Engineering, National Chung Cheng University, Chia-Yi 621, Taiwan; 5 Institute of Microbiology and Immunology, National Yang-Ming University, Taipei 112, Taiwan; 6 Department of Education and Research, Taichung Veterans General Hospital, Taichung 407, Taiwan; 7 Institute of Epidemiology, National Taiwan University, Taipei 100, Taiwan; 8 Institute of Bio-Pharmaceutical Sciences, National Yang-Ming University, Taipei 112, Taiwan; 9 Institute of Biotechnology in Medicine, National Yang-Ming University, Taipei 112, Taiwan; 10 Department of Computer Science and Information Engineering, National Taiwan University, Taipei 106, Taiwan

## Abstract

**Background:**

The development of microarrays permits us to monitor transcriptomes on a genome-wide scale. To validate microarray measurements, quantitative-real time-reverse transcription PCR (Q-RT-PCR) is one of the most robust and commonly used approaches. The new challenge in gene quantification analysis is how to explicitly incorporate statistical estimation in such studies. In the realm of statistical analysis, the various available methods of the probe level normalization for microarray analysis may result in distinctly different target selections and variation in the scores for the correlation between microarray and Q-RT-PCR. Moreover, it remains a major challenge to identify a proper internal control for Q-RT-PCR when confirming microarray measurements.

**Results:**

Sixty-six Affymetrix microarray slides using lung adenocarcinoma tissue RNAs were analyzed by a statistical re-sampling method in order to detect genes with minimal variation in gene expression. By this approach, we identified *DDX5 *as a novel internal control for Q-RT-PCR. Twenty-three genes, which were differentially expressed between adjacent normal and tumor samples, were selected and analyzed using 24 paired lung adenocarcinoma samples by Q-RT-PCR using two internal controls, *DDX5 *and *GAPDH*. The percentage correlation between Q-RT-PCR and microarray were 70% and 48% by using *DDX5 *and *GAPDH *as internal controls, respectively.

**Conclusion:**

Together, these quantification strategies for Q-RT-PCR data processing procedure, which focused on minimal variation, ought to significantly facilitate internal control evaluation and selection for Q-RT-PCR when corroborating microarray data.

## Background

Microarrays, by making use of the sequence resources created in genomic projects, are a powerful technology capable of measuring the expression levels of thousands of genes simultaneously and have dramatically expedited comprehensive understanding of gene expression profiles for disease development. For example, microarray technology has been used to compare gene expression profiles between normal and diseased cells and this has led to dramatic advances in the understanding of cellular processes at the molecular level [1]. Several microarray platforms are currently available. The short-oligonucleotide-based Affymetrix GeneChip^® ^arrays utilize multiple probes for each gene with an automated control for the experimental process from hybridization to quantification and thus provide reliable and comparable data [2]. The multiple probe sets for each gene are typically scattered across the surface of the Affymetrix microarrays. Variations in intensity from probe to probe or chip to chip for samples need to be resolved to obtain a reliable level of expression. Various statistical algorithms are available for probe-cell level normalization and expression-value summary.

Researchers are still confronted with challenging questions after completing the expression profiling and these include how to validate and standardize the data processing using proper statistical analysis. Quantitative-real time-reverse transcription PCR (Q-RT-PCR) is widely used and is a sensitive and robust technique for the detection and quantification of often rare mRNA targets [3]. Q-RT-PCR has also become one of the gold standards for both pathogen detection and gene expression studies and is the method of choice for corroborating microarray data [4]. In this study, the Q-RT-PCR system is based on the detection of the fluorescent activity and quantification of the TaqMan^® ^probe, which undergoes cleavage in proportion to the amount of PCR product formed [5,6]. By recording the amount of fluorescence emission at each cycle, it is possible to monitor the PCR reaction during the exponential phase where the first significant increase occurs and the amount of PCR product correlates to the initial amount of target template.

An appropriate internal control for Q-RT-PCR should be expressed stably across all data samples and if this is true, measurement of genes relative to the internal control will reflect the real gene expression. It implies that a reference gene should have a small variance and a sufficient intensity when applied as an appropriate internal control. Moreover, most published studies have focused on the identification of reference genes that can be used to normalize expression of a gene across patient samples or tissue types rather than within one specific type of tissue or cell line [7,8]. Generally speaking, housekeeping genes, such as *ACTB *(actin, β), *GAPDH *(glyceraldehyde-3-phosphate dehydrogenase), and 18S ribosomal RNA, are commonly employed in Q-RT-PCR analysis [9-11]. However, several studies have also demonstrated that the gene expression patterns of many commonly used internal controls may vary as a result of tissue type, experimental conditions or pathological state [12-15]. The "perfect" control gene for all Q-RT-PCR does not exist because variability in Q-RT-PCR data can also stem from differences in the expression of the reference gene, for example *GAPDH *and *ATCB*, on which the expression of all the other genes is based [16]. Although 18S ribosomal RNA has been shown to be a reliable control in many studies [7,8,17], it does not undergo reverse transcription when using oligo (dT) primers and is inappropriate for use when such primers are used. Szabo et al. developed statistical models to assist in identifying appropriate housekeeping genes as Q-RT-PCR normalization controls in one or multiple types of tissue samples [18]. However, their rigorous approach heavily relies on an assumption that there is a multivariate normal distribution for the microarray expression levels and may not fit a practical situation, especially without a large number of arrays. In addition, their models are only applicable to the analysis of random samples, not paired samples collected from each patient as in this study.

We aimed to address two unanswered questions associated with microarray target selections for Q-RT-PCR validation. Firstly, it is not certain which gene or genes can serve as better internal controls for Q-RT-PCR simply because there is no perfect internal control [15]. Secondly, a major challenge when scoring the correlation between microarray and Q-RT-PCR measurements remains unsettled because different probe level normalization methods may result in different correlations. In this study, we propose a statistical re-sampling method to display the variation pattern or to calculate the inter-quartile range (IQR) and the variance of gene expression levels that are associated with different probe level normalization methods. We utilized the block bootstrap re-sampling technique to circumvent the within-block dependence of Affymetrix microarray data when using paired adjacent normal and tumor samples from lung adenocarcinoma patients. Moreover, we employed box plot results for lung adenocarcinoma gene expression and identified *DDX5 *as a novel internal control for Q-RT-PCR. DDX5 is a highly conserved member of the DEAD box family and is known to be a RNA helicase that is involved in both pre-mRNA and pre-rRNA processing [19]. Twenty-three genes, which were differentially expressed between adjacent normal and tumor samples, were further selected for Q-RT-PCR analysis and were examined by microarray analysis with several probe level quantile normalization methods using either *DDX5 *or *GAPDH *as internal controls. No matter which probe level quantile normalization was used for comparison, *DDX5 *was a better internal control than *GAPDH *for lung cancer datasets.

## Results and Discussion

### Identification of a novel internal control through variation in gene expression levels

**Figure 1:**
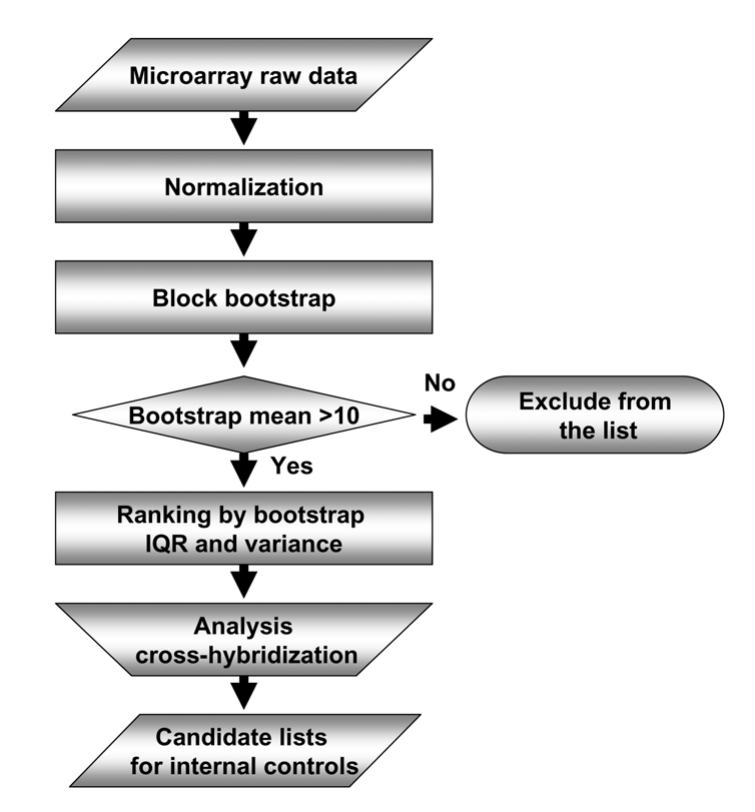
Flow chart for prioritization of potential internal controls.

**Figure 2:**
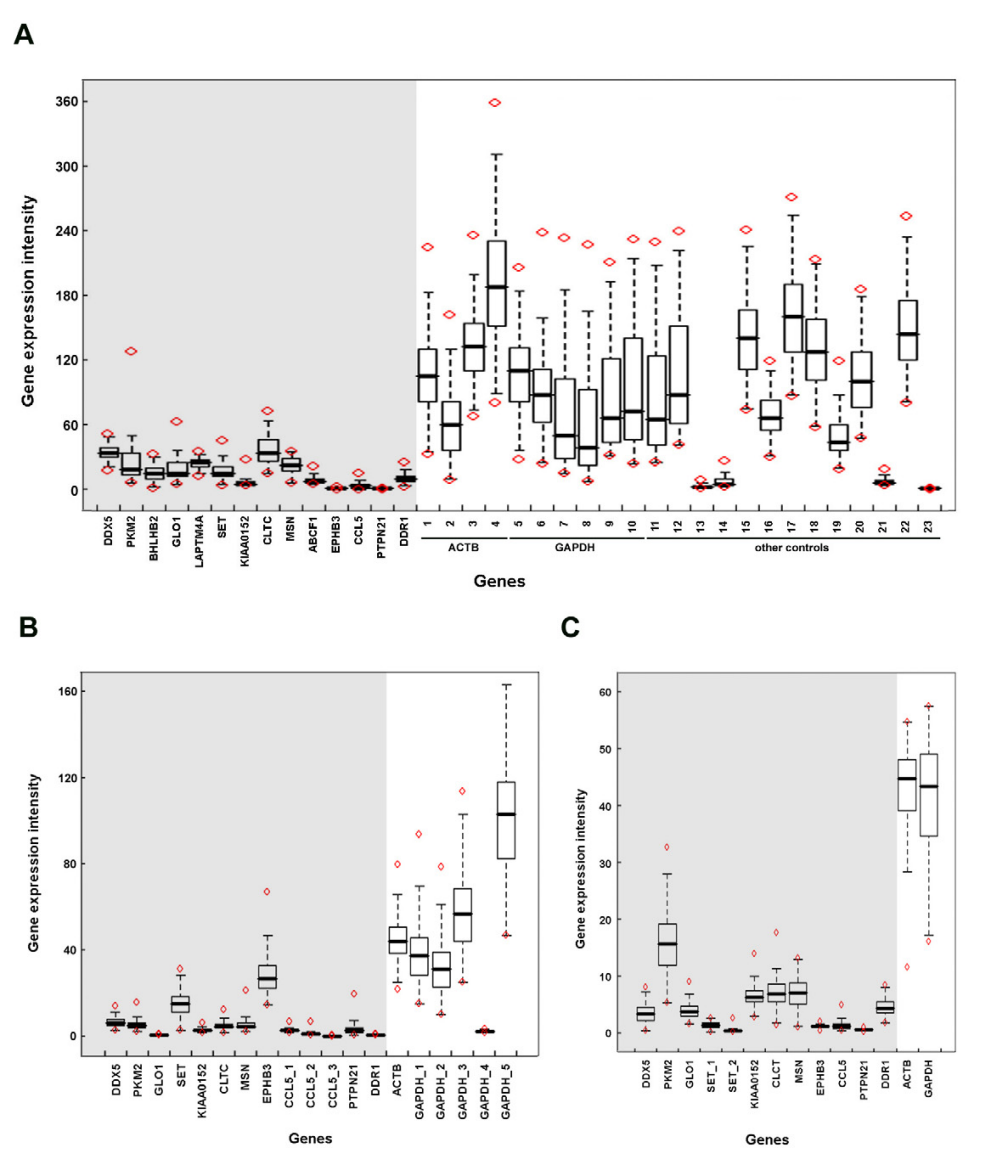
**Bootstrap box plots of the gene expression intensity of various internal controls**. (A) The box plot results show the best 14 internal control candidates, all of which exhibited consistent expression intensity in the NHRI lung adenocarcinoma microarray dataset for each re-sampling process. Moreover, also included are 10 well-known Q-RT-PCR internal controls contained in 23 probe sets on the HG-U133A chip. These are shown as #1–23 in x-axis. The detailed probe set characteristics were shown in Table 1. Except *ABCF1*, *BHLHB2 *and *LAPTM4A*, the gene expression intensities of top 12 internal control candidates, *GAPDH*, and *ACTB *from the Boston (B) and the Ann Arbor lung cancer datasets (C) were also compared. *DDX5*: (DEAD (Asp-Glu-Ala-Asp) box polypeptide 5), *PKM2*: (pyruvate kinase, muscle), *BHLHB2*: (basic helix-loop-helix domain containing, class B, 2), *GLO1*: (glyoxalase I), *LAPTM4A*: (lysosomal-associated protein transmembrane 4 alpha), *SET*: (SET translocation (myeloid leukemia-associated)), *CLTC*: (clathrin, heavy chain (Hc)), *MSN*: (MSN/ALK fusion; moesin/anaplastic lymphoma kinase fusion protein), *ABCF1*: (ATP-binding cassette, sub-family F (GCN20), member 1), *EPHB3*: (EPH receptor B3), *CCL5*: (chemokine (C-C motif) ligand 5), *PTPN21*: (protein tyrosine phosphatase, non-receptor type 21), *DDR1*: (discoidin domain receptor family, member 1), 1–4: *ACTB *(actin, beta), 5–6: *B2M *(beta-2-microglobulin), 7–12: *GAPDH *(glyceraldehyde-3-phosphate dehydrogenase), 13: HMBS (hydroxymethylbilane synthase), 14: *HPRT1 *(hypoxanthine phosphoribosyltransferase 1), 15–19: *RPL13A *(ribosomal protein L13a), 20: *RPL32 *(ribosomal protein L32), 21: *SDHA *(succinate dehydrogenase complex, subunit A, flavoprotein (Fp)), 22: *UBC *(ubiquitin C), 23: *YWHAZ *(tyrosine 3-monooxygenase/tryptophan 5-monooxygenase activation protein, zeta polypeptide).

Differentially expressed genes, identified by microarray through global normalization, require validation of their expression patterns through Q-RT-PCR, which generally employees one internal control for normalization. This distinct normalization method calls for a correct internal control for the microarray and Q-RT-PCR data comparison. To prioritize the potential internal controls for Q-RT-PCR analyses and to study the possibility of general utilization of these potential internal controls, we first applied the block bootstrapping technique to rank genes with variance and IQR (Fig. 1). This method was then used to evaluate the gene expression patterns of the various internal controls in a lung adenocarcinoma microarray dataset (referred to as NHRI dataset) with the goal to provide insights into which internal controls might be a best choice in this study. For illustration, we first used the RMA for probe-cell level normalization and expression-value summary of the 66 microarrays in the NHRI dataset. Associated with each gene, the appropriate averages of 1,000 bootstrap replicates for the 5 computed statistics produced a box plot that disclosed variation in gene expression. As a result, many potential internal controls were revealed. To prioritize the targets, we removed those with lower intensity signals and the multiple probe sets on Affymetrix chip with potential cross hybridization contamination between genes as determined by NetAffx™ Analysis Center [20]. The remaining 14 candidate internal controls, which exhibited small variance expression, were identified using the box plots and these were *DDX5*, *PKM2*, *BHLHB2*, *GLO1*, *LAPTM4A*, *SET*, *KIAA0152, CLTC*, *MSN*, *ABCF1*, *EPHB3*, *CCL5*, *PTPN21 *and *DDR1 *(Fig. 2A). In addition, the box plots for 10 well-known internal controls [15,21] (containing 23 probe sets) are also shown in Figure 2A. Surprisingly, 13 out of 23 probe sets have various levels of cross hybridization as identified by the NetAffx™ Analysis Center (Table 1). The unique characteristics of these well-known internal controls for Q-RT-PCR are their high expression levels and there are also huge variances.

The copy number of the individual housekeeping gene chosen for relative quantification should be in a similar range to that of the target gene to make comparative quantification possible [22]. Further analysis indicates that we can identify a series of internal control candidates, which have characteristics of small variance in different microarray intensity intervals (Additional file 1). These potential internal controls are presented in different intensity ranges in order to appropriately normalize different target genes. Despite the fact that these potential internal controls may exhibit a greater variance than *DDX5 *or other internal controls listed in Figure 2A, these potential internal controls have much smaller variance than *ACTB *and *GAPDH *(Additional file 1, right portion). This finding further supports a view that the intensities of normalized microarray data and the copy numbers of Q-RT-PCR detections in gene expression patterns could be examined in a similar range. Our approach may provide a method to identify potential internal controls to be in a similar range of expression as the selected target genes.

To prioritize the potential internal control for the lung cancer microarray data, two major public accessible lung cancer microarray datasets, which also used Affymetrix chips, namely the Boston and the Ann Arbor datasets [23,24], were included for data comparison. Eleven candidates, after the exclusion of *ABCF1*, *BHLHB2 *and *LAPTM4A*, were available in both Boston and Ann Arbor datasets and were included in the analysis. Figure 2B and 2C show the results of basic bootstrapping using these two datasets of unpaired design. All 11 candidates exhibited less variation than most well known internal controls, suggesting that all 11 candidates have potential to serve as an internal control, at least for lung cancer. To finalize the target for further empirical validation, these potential controls were sorted in the order of increasing gene expression intensities and decreasing IQR, respectively. As a result, *CLTC*, *DDX5 *and *MSN *were found to exhibit sufficient intensity. However, *DDX5 *gave the smallest variation among the three. Therefore, in this study, we chose *DDX5 *for further characterization.

**Figure 3:**
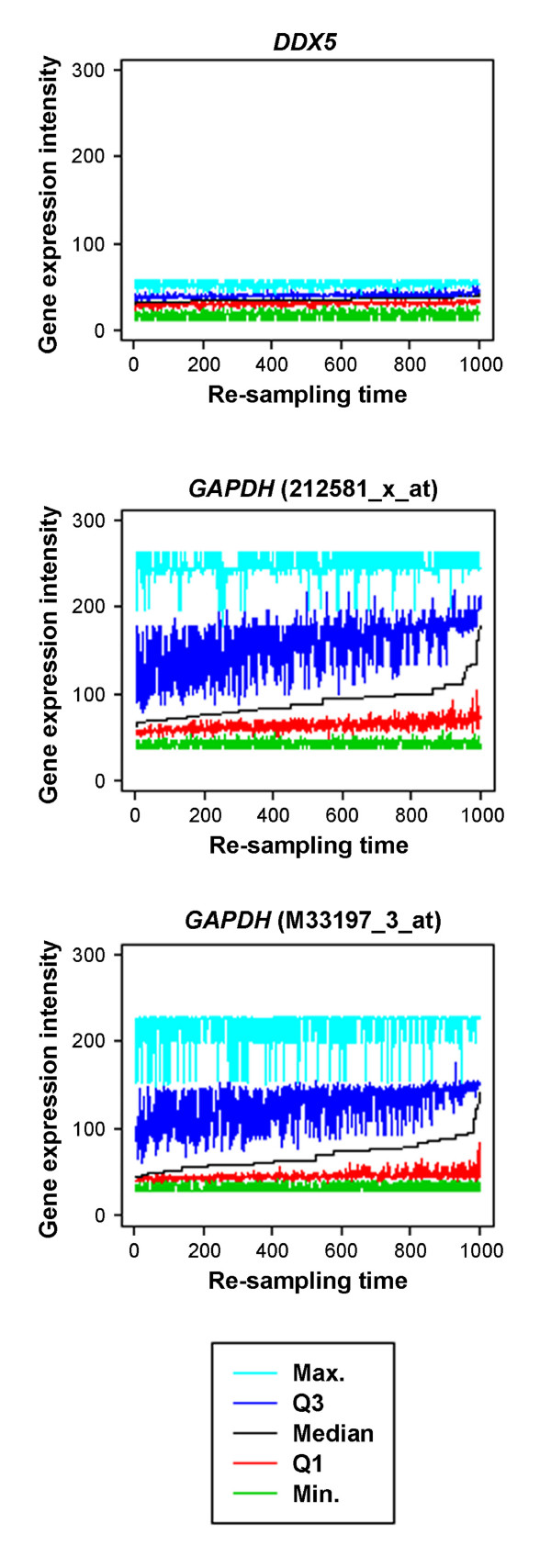
**Bootstrap replicates of the descriptive statistics related to the variation in gene expression**. In total, 27 pairs of adjacent normal and tumor samples with 12 un-paired samples were used in this analysis. A total of 39 blocks of microarray samples were used for the block bootstrap. The re-sampling process was repeated 1,000 times to obtain 1,000 bootstrap replicates of the minimum (green color), first quartile (red color), median (black color), third quartile (blue color) and maximum (cyan color) expression levels for each gene. Each result was ranked by the order of medians. The bootstrap replicates of all five statistics for *DDX5 *expression remain roughly constants, but those for *GAPDH *expression vary greatly.

Considering this study was a paired design with a moderate sample size, we applied the block bootstrapping technique and evaluated the gene expression patterns of various internal controls using the NHRI dataset (as described in Materials and Methods). A total of 39 blocks, resulting from 27 pairs of adjacent normal and tumor samples and 12 un-paired samples of microarrays, were used in the block bootstrap. The re-sampling process was repeated 1,000 times to obtained 1,000 bootstrap replicates of the minimum, first quartile, median, third quartile and maximum for the expression level of each gene. The bootstrap replicates for all five statistics for *DDX5 *expression are roughly constants, but those for *GAPDH *expression vary greatly (Fig. 3).

**Figure 4:**
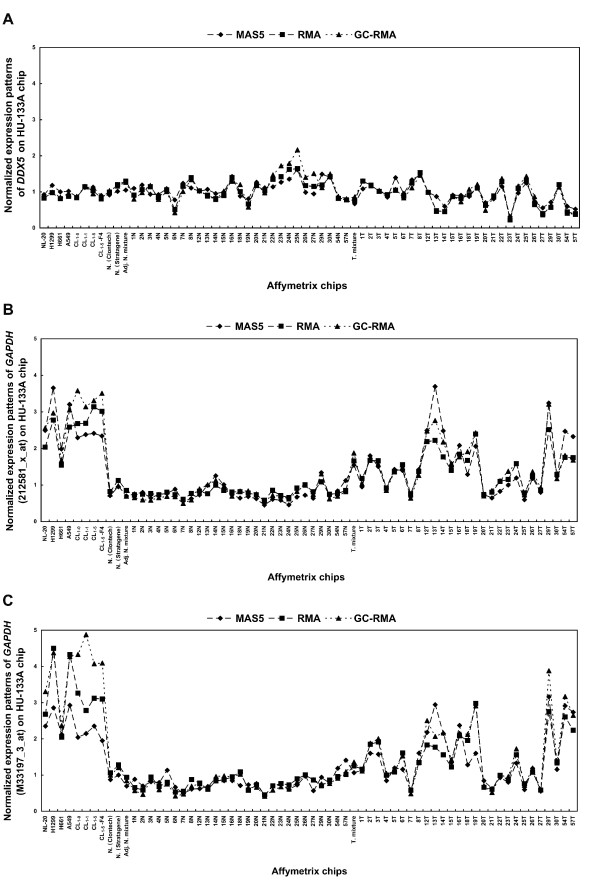
**Gene expression patterns of *DDX5 *and *GAPDH *from 66 Affymetrix microarray chips using three different probe level quantile normalizations**. The gene expression patterns of two internal controls, *DDX5 *(A) and *GAPDH *(B: 212581_x_at and C: M33197_3_at) from Affymetrix chips by MAS5 (marked by diamonds), RMA (marked by squares) and GC-RMA (marked by triangles) probe level quantile normalizations. Normalization was performed per chip and per gene using GeneSpring^® ^7.3 software. The expression levels of these two internal controls were related to the median of the intensities on the 66 chips. The *DDX5 *expression patterns in each chip did not significantly alter compared to greater variation in *GAPDH*.

*DDX5 *exhibited relatively similar expression patterns between adjacent normal and tumor samples as well as across various lung cancer cell lines when compared to *GAPDH *(Fig. 4). In addition to RMA, we also employed the other two different methods, MAS5 and GC-RMA, for the probe normalization to analyze 66 Affymetrix microarray chips using lung adenocarcinoma RNA samples. The three different normalization methods displayed consistent expression patterns for *DDX5*, suggesting that *DDX5 *may serve as a reliable internal control [25].

### Q-RT-PCR validation and comparison with microarray

We employed Affymetrix HG-U133A chips to elucidate the gene expression profiles for 27 pairwise primary lung adenocarcinomas. In total, 4437 out of 22283 differential expression transcripts between adjacent normal and tumor parts were clustered by the Wilcoxon signed-rank test with *p*-value (*p *< 0.01) adjusted for multiple genes in terms of the false discovery rate [26]. By examining 24 paired lung adenocarcinoma samples with Q-RT-PCR, the results from 23 genes were subject to statistical analysis. Two internal controls, *DDX5 *and *GAPDH*, were used to obtain the relative expression patterns and the results compared. The correlation between microarray with RMA normalization and Q-RT-PCR were analyzed by Pearson's and Kendall's τ correlations and the summary is shown in Table 2. Based on Pearson's correlation coefficient, the percentage of genes with significant correlations between the microarray and Q-RT-PCR expression was 70% (16 out of 23) using *DDX5 *normalization, which is much higher than the 48% (11 out of 23) for *GAPDH*. If we only focus on the significant Q-RT-PCR expressions (using *DDX5 *as an internal control) based on tumor *vs. *adjacent normal, the percentage of genes with similar patterns between microarray and Q-RT-PCR expression was 91% (21 out of 23). Similar results were also observed using Kendall's τ correlation coefficient (Table 2). To address whether a good internal control for Q-RT-PCR was able to reflect the gene expression pattern in microarray, differentially expressed genes identified by the Wilcoxon signed-rank test with *p *values ranging from 10^-2 ^to 10^-6 ^were compared with the Q-RT-PCR results. Again, a percentage around 80% of significant correlation was found when *DDX5 *was used as an internal control.

### The expression pattern comparisons of DDX5 in other microarray datasets

**Figure 5:**
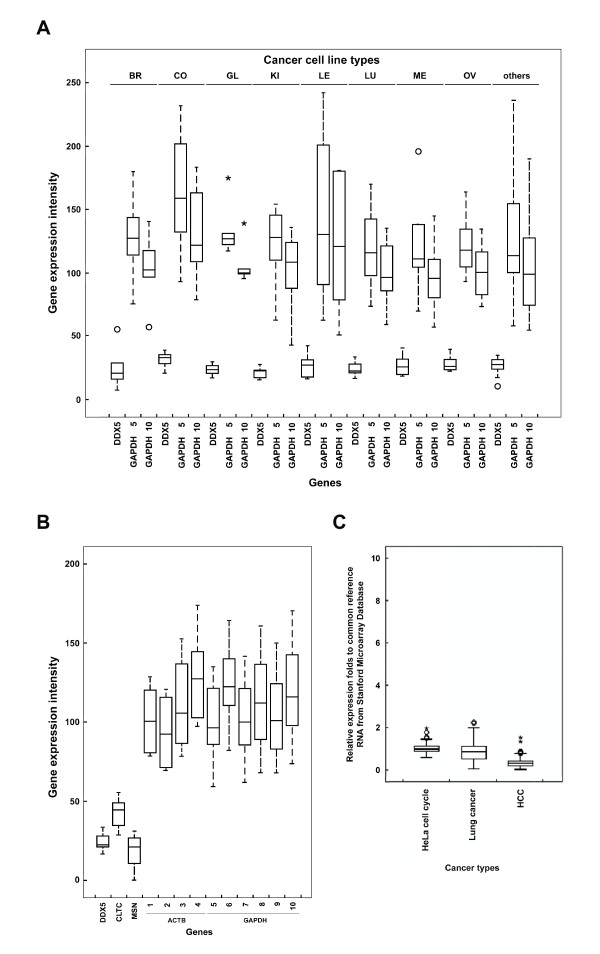
**Box plots of *DDX5 *relative expression patterns exhibit small variation across various microarray datasets**. The gene expression patterns of *DDX5 *were obtained from other microarray databases. These datasets were from 84 cancer cell lines (NCI60), which were classified into 8 different cancer cell types and other cell lines (A). *GAPDH #5*: M33197_3_at; *GAPDH #10*: 212581_x_at. The box plot results indicated that *DDX5 *exhibited only small variation across the various NCI60 cell types. For the lung cancer cell lines, *DDX5*, *CLTC *and *MSN *all gave lower variances than *ACTB *and *GAPDH *(B). Both *ACTB *(#1–4) and *GAPDH *(#5–10) are contained in 10 probe sets on the HG-U133A chip and are shown as #1–10 on x-axis and in Table 1. The variation of *DDX5 *was also smaller for the HeLa cell cycle dataset, lung cancer dataset and HCC dataset from the Stanford Microarray Database (C). BR: breast cancer, CO: colon cancer, GL: glioblastoma, KI: Kidney, LE: leukemia, LU: lung cancer, ME: melanoma and OV: ovarian cancer.

To explore the possibility that *DDX5 *can be generally utilized as an internal control, we further analyzed *DDX5 *expression patterns using a new Affymetrix based NCI60 dataset [27] from Genomics Institute of the Novartis Research Foundation (GNF). The expression patterns of *DDX5 *were investigated in 8 different types of cancer cell lines (NCI60) and several additional cell lines with a total of 84 cell lines and *DDX5 *exhibited much smaller variation than *GAPDH *for all cell types (Fig. 5A). Moreover, the expression patterns of *DDX5*, *CLTC *and *MSN *(Fig. 5B) were further compared with *ACTB *and *GAPDH *in the lung cancer cell lines found in NCI60. All three candidates showed less variance than both well-known internal controls. Similar patterns were also observed in our NHRI dataset (Fig. 2A). Additional cDNA microarray datasets were also downloaded from Stanford Microarray Database [28], including lung cancers [29], hepatocellular carcinomas (HCC) [30] and the HeLa cell cycle dataset [31], which reported cell cycle related genes by synchronizing HeLa cells at different phases of cell cycle [32] (Fig. 5C). Based on these comparisons, we conclude that *DDX5 *is evidently a novel internal control with a relatively constant expression pattern in lung adenocarcinoma. In addition, using limited microarray datasets analyzed in this paper, the variation in *DDX5 *levels was also relatively small in other cancer cell lines, raising the possibility that *DDX5 *could serve as a novel internal control.

## Conclusion

In summary, we adapted block bootstrap for using with a paired design to circumvent the within-pair dependence. This proposed re-sampling strategy together with the use of a box plot provides a useful distribution-free statistical procedure for exploratory data analysis. This systematic analysis procedure focused on identifying genes with minimal variation in their microarray data, which facilitates the essential internal control selection steps prior to Q-RT-PCR analysis. Finally, systematic microarray and Q-RT-PCR analyses reveal that the proposed re-sampling technique of block bootstrap suits paired design experiments and adequately detects genes with minimal variation in a microarray dataset.

## Methods

### Sample preparation

A total of 66 samples were used for microarray analysis, including paired adjacent normal-tumor samples from 27 patients underwent surgery for lung cancer at the Taipei Veterans General Hospital, two tissue mixtures from the Taichung Veterans General Hospital (one was adjacent normal lung mixtures and the other was lung adenocarcinoma mixtures), two commercial human normal lung tissues (Clontech (Catalog No. 636524) and Stratagene (Catalog No. 735020)), one immortalized, nontumorigenic human bronchial epithelial cell line (NL-20 (ATCC^® ^No. CRL-2503™)) and 7 lung cancer cell lines (A-549 (ATCC^® ^No. CCL-185™), NCI-H1299 (ATCC^® ^No. CRL-5803™), NCI-H661 (ATCC^® ^No. HTB-183™), CL_1-0 _[33], CL_1-1 _[33], CL_1–5 _[33], and CL_1–5_-F4 [34]).

### RNA extraction and reverse transcription

We used the total RNA samples for Q-RT-PCR analyses. RNA preparation and analysis were performed according to the previous study [11]. Briefly, the quality of the total RNA for microarray analysis was determined using Spectra Max Plus (Molecular Devices) and had an OD260/OD280 ratio ranging from 1.9 to 2.1. RNA was subjected to reverse transcription with random hexamer primers. To hydrolyze contaminating DNA in the RNA preparations, RNA was incubated with amplification-grade DNase I (Life Technologies, Gaithersburg, MD). After incubating the reaction mixture, the reaction was stopped by heating at 65°C. After DNase treatment, the RNA was subjected to reverse transcription reaction by the ThermoScript™ RT-PCR system (Life Technologies) and cDNAs were then used in the Q-RT-PCR.

### Q-RT-PCR

Q-RT-PCR was used to measure the mRNA expression levels of various differentially expressed genes between adjacent normal and lung tumors by using 384-well plates (ABI PRISM 7900 HT Sequence Detection System). The cDNAs were served as templates (diluted 200×) for Q-RT-PCR by using TaqMan^® ^Universal PCR Master Mix kit (Applied Biosystems, Foster City, CA). Each 10 μl of quantitative PCR reaction mixture contained 5 μl of 2× TaqMan^® ^Universal Master Mixture (Applied Biosystems), 4 μl of 200× diluted cDNA product mixture, and 1 μl of PCR forward and reverse primers and probe. To standardize the quantification of the selected target genes, *DDX5 *and *GAPDH *served as internal controls and were quantified on the same plate as the target genes. The cycle threshold (CT) value of *DDX5 *or *GAPDH *was used to normalize the target gene expression values, referred to as the ΔCT, which was used to adjust differences among samples. Approximately 50 genes were selected as the start of this study. These genes were subjected to Q-RT-PCR analysis under 200× diluted cDNA conditions due to insufficient cDNA for large-scale screening. The reaction products by agarose gel electrophoresis showed that many of the amplifications contained no detectable PCR product. If the Q-RT-PCR reactions failed in 50% of the assays, the data were excluded from further statistical analysis. In addition, several gene products exhibited multiple bands when examined on the agarose gels. Even though we used the TaqMan system, we were not comfortable with such data and these were also discarded. The Assays-on-Demand IDs (Applied Biosystems) for the 25 genes are shown in Table 3.

### Microarray experiments, normalization and Wilcoxon signed-rank test

Protocols, reagents for hybridization, washing and staining followed previous methods [35] and the Affymetrix's instructions [36]. Labeled cDNA was hybridized to the Affymetrix GeneChip Test 3 Array to verify quality prior to hybridize to the Affymetrix Human Genome U133A Array. The data discussed in this publication have been deposited in NCBIs Gene Expression Omnibus (GEO) [37] and are accessible through GEO Series accession number GSE7670. The images were transformed into text files as intensity information using the MAS5.0 (Microarray Suite software 5.0) developed by Affymetrix [38,39]. We used three array processing methods to produce and normalize the Affymetrix expression signals for the transcripts based on corresponding probe pairs of oligonucleotides. MAS5.0 as provided by Affymetrix was used to carry out the probe-pairs adjustment. Both MAS5.0 and RMA [40], Robust Multichip Average via quantile adjustment, are commonly used. Another algorithm GC-RMA [41] is similar to RMA, but pools the probes with comparable numbers of G-C bonds to achieve a stable mismatch adjustment. Based on paired adjacent normal and tumor samples of 27 patients, we used the Wilcoxon signed-rank test [42] to identify differentially expressed genes. In contrast to Student's *t*-test inappropriately used in some studies [43], the signed-rank test is distribution-free and adjusts for the paired design.

### Block bootstrapping

To assess the variation in the microarray expression level for a specified gene in an experiment of moderate sample size and possibly including paired samples, we designed a block bootstrapping procedure to analyze the microarray data from 66 lung samples, containing 27 pairs of patient adjacent normal-tumor samples and 12 un-paired samples. Bootstrapping is a breakthrough statistical approach using a computationally intensive re-sampling technique and it allows complex problems to be solved in which the accuracy of a devised statistical procedure can not be analytically evaluated [44,45]. Block bootstrapping was originally named for re-sampling methods in dependent cases, especially time series data [46]. The basic bootstrap generates artificial samples that allow the making of an inference of interest through re-sampling the original data with replacement in which all observations are assumed to be mutually independent and from the same distribution. To guarantee the structure of independence in bootstrap re-sampling, we employed the concept of blocking for the paired data by treating each individual patient, mixture tissue or cell line as a block. By selecting an observation within the block with equal probability when combined with all the other un-paired samples, we obtained an independently re-sampled dataset. We then created a bootstrap sample by randomly sampling the blocks in the dataset, and computed the bootstrap replicates of the relevant summary statistics of the expression levels. Repeating this bootstrap re-sampling scheme sufficient times, such as 1,000, we then used the averages of these bootstrap replicates to reveal the variation in expression summaries corresponding to a specific gene across the microarrays. Appropriate internal controls can be selected by ranking the variations in gene expression.

### Correlations of microarray and Q-RT-PCR data

To explicate the correlation between microarray and Q-RT-PCR in this study of paired design, we calculated the differences between the log-scaled measurements of the Q-RT-PCR and microarraydata from the tumor and adjacent normal tissues of 24 patients. The other 3 paired samples did not have sufficient materials for further Q-RT-PCR analysis. Pearson's and Kendall's τ correlation coefficients were then tested at a significant level of 0.05. Summarization of the expression levels and normalization for microarray data were conducted using GeneSpring^® ^7.3 (Silicon Genetics, Redwood City, CA). The computer programs for the block bootstrapping method and correlation using R 2.1.1 [47] are presented in the Additional File 2.

## Authors' contributions

LJS carried out the quality control of microarray and Q-RT-PCR studies, participated in the data mining and wrote the manuscript. CWC performed the statistical analysis and wrote the manuscript. KCC and CJL helped to analyze the DNA microarray results. SCL performed the Q-RT-PCR experiments. YCW, CHL, JWP and SLH provided the specimens for microarray analysis and partial grant supports. CHC and CYH designed this study, had the responsibilities for the budget supports and wrote the manuscript. All authors read and approved the final manuscript.

## Supplementary Material

Additional File 1 A series of potential internal controls with relatively small variance in different microarray intensity intervals. The following potential internal controls have the characteristics of small variance in different microarray intensity intervals, including *SKP1A *(S-phase kinase-associated protein 1A (p19A)) (intensity range: 40 to 50), *OAZ1 *(ornithine decarboxylase antizyme 1) (50 to 60), *H3F3A *(H3 histone, family 3A) (60 to 70), *RPL37 *(ribosomal protein L37) (70 to 80), *RPS15A *(ribosomal protein S15a) (80 to 90) and *RPS4X *(ribosomal protein S4, X-linked) (large than 90). The relative expression patterns of *ACTB *and *GAPDH *were also shown on the right portion for comparison. These panels of different intensity genes may be considered as alterative internal candidates for Q-RT-PCR. Click here for file

Additional File 2 Re-sampling method for balanced block design data. Apply bootstrapping method to balanced block design data. Click here for file

## Figures and Tables

**Table 1:** Summary of probe set characteristics of 10 well-known internal controls for Q-RT-PCR in Affymetrix HG-U133A chip

**Gene symbol**	**Probe set ID**	**Cross-hybridization**	**Column # in Fig. 2A**
ACTB	AFFX-HSAC07/X00351_M_at	no	1
	AFFX-HSAC07/X00351_5_at	no	2
	AFFX-HSAC07/X00351_3_at	no	3
	200801 _x at	yes	4
GAPDH	AFFX-HUMCAPDH/M33197_M_at	no	5
	AFFX-HUMGAPDH/M33197_5_at	no	6
	AFFX-HUMGAPDH/M33197_3_at	no	7
	217398_x_at	yes	8
	213453_x at	yes	9
	212581_x_at	yes	10
B2M	216231_s_at	yes	11
	201891_s_at	yes	12
HMBS	203040_s_at	no	13
HPRT1	202854_at	no	14
RPL13A	212790_x_at	yes	15
	211942_x_at	yes	16
	210646_x_at	yes	17
	200716_x_at	yes	18
	200715_x at	yes	19
RPL32	200674_s_at	no	20
SDHA	201093_x_at	yes	21
UBC	211296_x_at	yes	22
YWHAZ	214848_at	no	23

**Table 2:** Summary of the correlations between microarray and Q-RT-PCR analyzed by Pearson's and Kendall's τ correlations.

**Gene**	**Pearson's Correlation**		**Kendall's τ Correlation**	
	
	**DDX5**	**GAPDH**	**DDX5**	**GAPDH**
ASK	0.13	-0.16	0.14	-0.19
BUB1B	0.53	0.47	0.39	0.33
CDCA8	0.68*	0.49	0.52*	0.36
CENTD2	0.65**	0.16	0.50**	0.05
CXCL5	0.62**	0.60**	0.39**	0.34*
CYP27A1	0.30	0.53**	0.19	0.41**
FLJ10540	0.70**	0.35	0.50**	0.16
FLJ20530	0.54**	-0.14	0.40**	-0.08
FLJ20605	0.03	0.25	0.01	0.18
FY	0.47*	0.52*	0.30*	0.34*
FZD4	0.45*	0.42	0.28	0.25
GARP	0.55*	0.53*	0.24	0.26
MMP9	0.70**	0.64**	0.54**	0.49**
MSR1	0.79**	0.79**	0.50**	0.57**
PA26	0.56**	0.68**	0.35*	0.49**
S100A2	0.67**	0.57**	0.42**	0.38**
SERPINA3	0.70**	0.49	0.49*	0.41*
SOX4	0.11	0.14	-0.06	-0.03
SRD5A1	0.69**	0.61**	0.50**	0.37*
T1A-2	0.31	0.19	0.22	0.13
TEK	0.41	0.47*	0.24	0.32*
TOPK	0.58**	0.28	0.39*	0.16
TROAP	0.60**	0.46*	0.40**	0.30*

*: *P *< 0.05, **: *P *< 0.01.

**Table 3:** Summary table of Assays-on-Demand ID of 23 genes and 2 internal controls for Q-RT-PCR.

**Genes**	**Description**	**Assays-on-Demand ID**
ASK	activator of S phase kinase	Hs00272696_m1
BDB1B	BUB1 budding uninhibited by benzimidazoles 1 homolog beta (yeast)	Hs00176169_m1
CDCA8	cell division cycle associated 8	Hs00216479_m1
CKNTD2	centaurin, delta 2	Hs00362929_m1
CXCL5	chemokine (C-X-C motif) ligand 5	Hs00171085_m1
CYP27A1	cytochrome P450, family 27, subfamily A, polypeptide 1	Hs00168003_m1
FLJ10540	chromosome 10 open reading frame 3	Hs00216688_m1
FLJ20530	hypothetical protein FLJ20530	Hs00215334_m1
FLJ20605	hypothetical protein FLJ20605	Hs00215486_m1
FY	Duffy blood group	Hs00266671_s1
FZD4	frizzled homolog 4(Drosophila)	Hs00201853_m1
GARP	glycoprotein A repetitions predominant	Hs00194136_m1
MMP9	matrix metalloproteinase 9 (gelatinase B, 92 kDa gelatinase, 92 kDa type IV collagenase)	Hs00234579_m1
MSR1	macrophage scavenger receptor 1	Hs00234007_m1
PA26	sestrin 1	Hs00205427_m1
S100A2	S100 calcium binding protein A2	Hs00195582_m1
SERPINA3	serine (or cysteine) proteinase inhibitpr, clade A (alpha-1 antiproteinase, antitrypsin), member 3	Hs00153674_m1
SOX4	SRY(sex determining region Y)-box 4	Hs00268388_s1
SRD5A1	steroid-5-alpha-reductase, alpha polypeptide 1 (3-oxo-5 alpha-steroid delta 4-dehydrogenase	Hs00602694_mH
	alpha 1)	
T1A-2	lung type-I cell membrane-associated glycoprotein	Hs00366764_m1
TEK	TEK tyrosine kinase, endothelial (venous malformations, multiple cutaneous and mucosal)	Hs00176096_m1
TOPK	T-LAK cell-originated protein kinase	Hs00902988_m1
TROAP	trophinin associated protein (tastin)	Hs00193896_m1
DDX5	DEAD (Asp-(Glu-Ala-Asp) box polypeptide 5	Hs00189323_m1
GAPDH	glyceraldehyde-3-phosphate dehydrogenase	Hs99999905_m1
