# Supplementary material for: Selection of DDX5 as a novel internal control for Q-RT-PCR from microarray data using a block bootstrap re-sampling scheme
Source: BMC Genomics. 2007 Jun 1;8:140. doi: 10.1186/1471-2164-8-140 (PMC1894975; doi:10.1186/1471-2164-8-140)
Supplement: Additional File 1 — A series of potential internal controls with relatively small variance in different microarray intensity intervals. The following potential internal controls have the characteristics of small variance in different microarray intensity intervals, including SKP1A (S-phase kinase-associated protein 1A (p19A)) (intensity range: 40 to 50), OAZ1 (ornithine decarboxylase antizyme 1) (50 to 60), H3F3A (H3 histone, family 3A) (60 to 70), RPL37 (ribosomal protein L37) (70 to 80), RPS15A (ribosomal protein S15a) (80 to 90) and RPS4X (ribosomal protein S4, X-linked) (large than 90). The relative expression patterns of ACTB and GAPDH were also shown on the right portion for comparison. These panels of different intensity genes may be considered as alterative internal candidates for Q-RT-PCR. [file 1471-2164-8-140-S1.doc]

**Additional file 1 – A series of potential internal controls with relatively small variance in different microarray intensity intervals.**


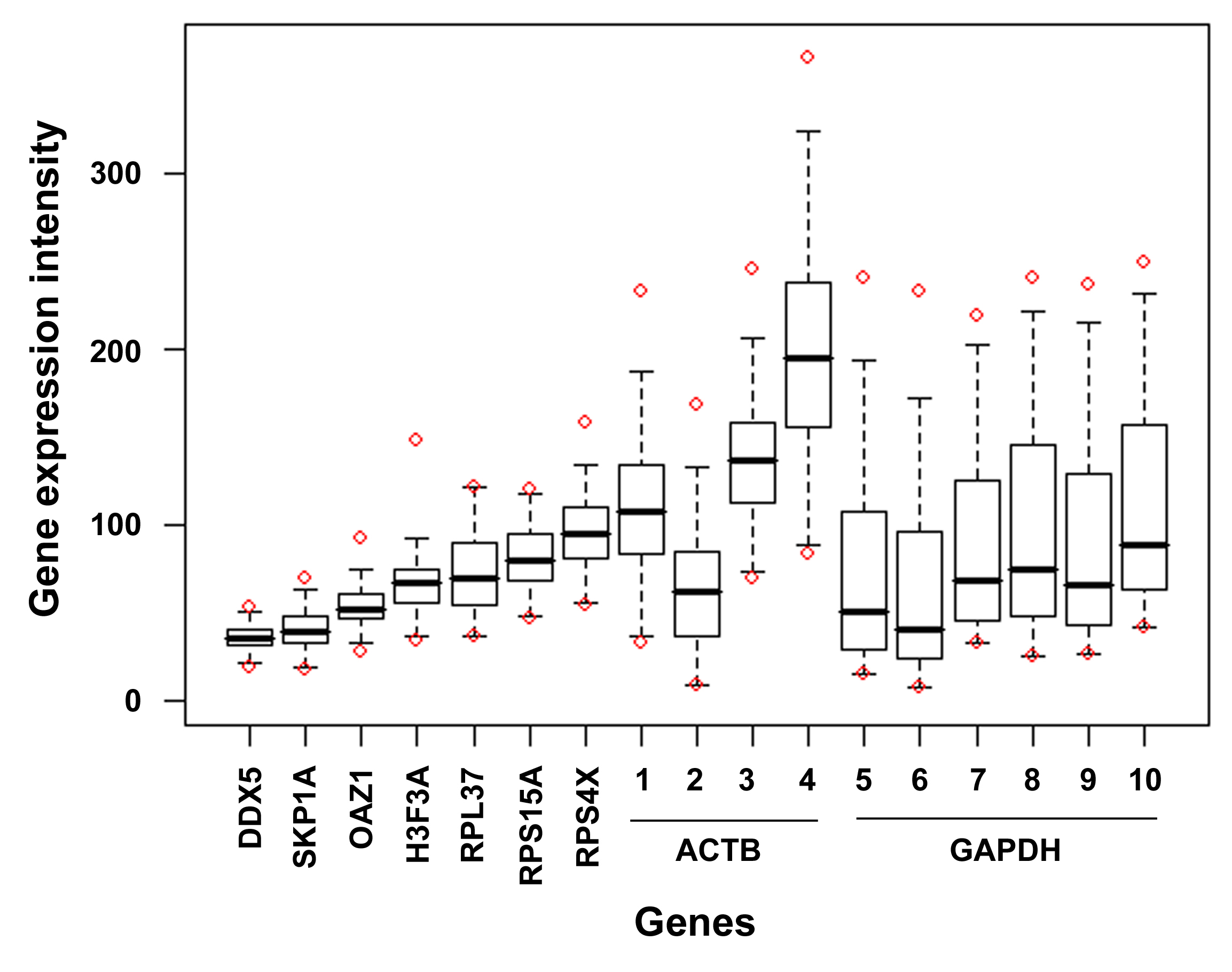


The following potential internal controls have the characteristics of small variance in different microarray intensity intervals, including *SKP1A* (S-phase kinase-associated protein 1A (p19A)) (intensity range: 40 to 50), *OAZ1* (ornithine decarboxylase antizyme 1) (50 to 60), *H3F3A* (H3 histone, family 3A) (60 to 70), *RPL37* (ribosomal protein L37) (70 to 80), *RPS15A* (ribosomal protein S15a) (80 to 90) and *RPS4X* (ribosomal protein S4, X-linked) (large than 90). The relative expression patterns of *ACTB* and *GAPDH* were also shown on the right portion for comparison. These panels of different intensity genes may be considered as alterative internal candidates for Q-RT-PCR.
