# Supplementary material for: Selection of DDX5 as a novel internal control for Q-RT-PCR from microarray data using a block bootstrap re-sampling scheme
Source: BMC Genomics. 2007 Jun 1;8:140. doi: 10.1186/1471-2164-8-140 (PMC1894975; doi:10.1186/1471-2164-8-140)
Supplement: Additional File 2 — Re-sampling method for balanced block design data. Apply bootstrapping method to balanced block design data. [file 1471-2164-8-140-S2.doc]

**Additional file 2 – Re-sampling method for balanced block design data**

##################################################################

## Re-sampling method for balanced block design data ##

## ##

##PROGRAM: blockboot ##

## ##

##PURPOSE: Apply bootstrapping method to balanced block design data ##

## ##

## INPUT: ##

## pairdata: A p by n microarray data matrix with p genes in n arrays for block ##

## design, the names of genes and arrays should be specified as row ##

## name and column name. The order of n arrays should be by block. ##

## otherdata: A p by n microarray data matrix with p genes in n arrays for ##

## independent samples, the names of genes and arrays should be ##

## specified as row name and column name. This dataset can be ##

## ignored. ##

## r: The block size ##

## m: The total number of replicates ##

## geneid: A vector of the names for the target genes ##

## ##

## OUTPUT: ##

## matrix of averages of quantiles for each gene: min, lower whisker, ##

## the lower hinge, the median, the upper hinge, the upper whisker, ##

## and the max. ##

##################################################################

# re-sampling method for balanced block design data

blockboot=function(pairdata, otherdata=NULL, r, m, geneid){

n=length(geneid)

B=(dim(pairdata)[2])/r

sumstat=array(0, c(7,m,n))

id=rep(0,B)

z=NULL

# re-sampling function

resample <- function(x, size, ...)

if(length(x) <= 1) { if(!missing(size) && size == 0) x[FALSE] else x

} else sample(x, size, ...)

# re-sampling

for(i in 1:m){

for(j in 1:B){

id[j]=resample(c((r*j-(r-1)): (r*j)), 1)

}

id1=as.numeric(id)

pdata=pairdata[, id1]

if(!is.null(otherdata)){

alldata=cbind(pdata, otherdata)}else{alldata=pdata}

rownames(alldata)=row.names(pdata)

xx=c(1:(ncol(alldata)))

bootx=sample(xx, ncol(alldata) , replace = TRUE)

redata=alldata[geneid, bootx]

# calculate quantiles

r1=data.frame(t(redata))

sumstat[2:6,i,]=boxplot(r1, plot=FALSE)$stats

sumstat[1,i,]=apply(r1, 2, min)

sumstat[7,i,]=apply(r1, 2, max)

}

# create output file

for(k in 1:n){

zz=apply(sumstat[,,k], 1, mean)

z=cbind(z, zz)

}

z1=t(z)

rownames(z1)=row.names(redata)

outz=t(z1)

pp=c("min","lower whisker","lower hinge","median","upper hinge", "upper whisker","max")

row.names(outz)=pp

outz

}

**Example:**

Assume the file name of paired data is pair.txt, and the file name of independent samples is other.txt.

Program:

x=read.table("c://pair.txt", header=T)

y=read.table("c://other.txt", header=T)

geneid=c("200033_at","222143_s_at","212882_at","200673_at","202360_at")

blockboot(pairdata=x, otherdata=y, r=2, m=100, geneid=geneid)

Result:

|  | 200033_at | 222143_s_at | 212882_at | 200673_at | 202360_at |
| --- | --- | --- | --- | --- | --- |
| min | 17.9874 | 2.1857 | 1.364627 | 12.73703 | 2.902293 |
| lower whisker | 21.49145 | 2.564185 | 1.633893 | 16.08557 | 3.257504 |
| lower hinge | 31.00075 | 3.251361 | 2.121959 | 22.24929 | 4.105166 |
| median | 35.75543 | 3.550099 | 2.359269 | 25.10806 | 4.647467 |
| upper hinge | 40.51234 | 3.920274 | 2.626583 | 28.10451 | 5.117955 |
| upper whisker | 50.57359 | 4.514321 | 3.098999 | 33.39878 | 6.244111 |
| max | 53.18967 | 4.573681 | 3.355412 | 36.0994 | 6.847305 |
